# Identification of candidate genes for myeloma-induced osteocyte death based on microarray data

**DOI:** 10.1186/s13018-016-0411-0

**Published:** 2016-07-12

**Authors:** Honglai Tian

**Affiliations:** Department of Orthopaedics, Qilu Hospital of Shandong University, No. 42 Wenhua West Road, Jinan, 250012 China

**Keywords:** Multiple myeloma, Osteocyte, Differentially expressed genes, Enrichment analysis, Protein-protein interaction network

## Abstract

**Background:**

The study was aimed to investigate the molecular mechanisms of osteocyte death in multiple myeloma (MM) patients.

**Methods:**

GSE27372 was downloaded from Gene Expression Omnibus, including three HOB-01 (osteocyte cell line) control samples and three HOB-01 samples co-cultured with JJN3 (human MM cell line). After the differentially expressed genes (DEGs) were identified by Student’s *t* test method, enrichment analyses were performed for them using DAVID software. Using TRANSFAC, TSGene, and tumor-associated gene (TAG) databases, functional annotation was conducted for the DEGs. Additionally, protein-protein interaction (PPI) network and sub-network analyses were performed using STRING database and Cytoscape software.

**Results:**

Total 393 DEGs were identified, including 22 transcription factors (e.g., *KLF4* and *IRF8*) and 37 TAGs. Enrichment analysis suggested that *EGF*, *S1PR1*, and *NPY1R* were enriched in the function of circulatory system development. EGF (degree = 31) and EGR1 (degree = 19) had high degrees and interactions in the PPI network. In the sub-network, S1PR1, C3AR1, and NPY1R could interact with each other.

**Conclusions:**

These DEGs might participate in the osteocyte apoptosis induced by myeloma cells. These findings might provide a theoretical basis for a better understanding of the osteolysis in MM patients.

## Background

Multiple myeloma (MM) originates in neoplastic plasma cell disorder, and it is characterized by the clonal proliferation of malignant plasma cells in the bone marrow. As the second most general hematological cancer, the incidence of MM worldwide is about 1.5/100,000 new case [1]. It is also found that the incidence of MM is higher in men than in women, as well as in black than white in the USA [2]. MM is always associated with organ dysfunction, especially osteolysis [3]. Although the survival rate of MM patients is increased due to the advanced medicines [4], MM continues to be considered as an incurable disease, and further study is required to fully understand the potential molecular mechanisms of osteolysis in MM patients.

To adopt a volume appropriate for the local environment, the bone continuously remodels to keep the balance between bone formation and resorption mediated by osteocytes, osteoblasts, and osteoclasts [5]. Osteocyte represents the most abundant cell type in the bone and is actively involved in bone turnover. Dickkopf 1(*DKK1*) levels increase in the peripheral blood and bone marrow plasma of MM patients and relate to the development of osteolytic lesions [6, 7]. Tumor necrosis factor-related apoptosis-inducing ligand (*TRAIL*) produced by myeloma cells is positively correlated with osteolytic markers (such as urinary deoxypyridinoline and serum calcium), indicating that *TRAIL* may function in osteolysis of MM patients [8]. Via promoting the expression of receptor activator of NF-kB (*RANK*) in osteoclast precursors, c-Akt (*AKT*) plays a role in the osteoclast formation and bone osteolysis induced by MM [9]. Giuliani et al. found that MM cells promoted osteocyte death and altered the transcriptional profile in osteocyte [10]. However, they did not further perform comprehensive bioinformatics analysis to investigate the internal causes. In spite of the above researches, the molecular mechanism of the increased osteocyte death in MM patients is still unclear.

In our study, in order to investigate the molecular mechanism of osteocyte death in MM patients, we reanalyzed the gene expression profile in Giuliani et al. study and identified the differentially expressed genes (DEGs) between normal osteocytes and osteocytes affected by MM cells. The enriched functions and pathways of DEGs were further identified. In addition, we constructed the protein-protein interaction (PPI) network of DEGs. Then, the most significant sub-network was screened out.

## Methods

### Affymetrix microarray data

Gene transcriptional profile of GSE27372 [10] was downloaded from Gene Expression Omnibus (GEO) database (https://www.ncbi.nlm.nih.gov/geo/), and GSE27372 was based on the platform of GPL570 [HG-U133_Plus_2] Affymetrix Human Genome U133 Plus 2.0 array. There were six specimens in this dataset, including three HOB-01 (osteocyte cell line) control samples and three HOB-01 samples co-cultured with JJN3 (human myeloma cell line).

### DEGs screening

AFFY package (version 1.28.0, http://www.bioconductor.org/packages/release/bioc/html/affy.html) of Bioconductor [11] was used to pre-process the Affymetrix microarray data. All raw data were scaled according to the robust multi-array average (RMA) method [12] with default settings. After background correction, quantile normalization, and probe summarization, the gene expression matrix was obtained. The Student’s *t* test method [13] was adopted to analyze the expression differences between control and co-culture group. For each significant DEG, both *P* value <0.05 and |log_2_ fold change (FC)| >0.58 need to be met.

### Function and pathway enrichment analyses of DEGs

Functional annotation for DEGs was performed by Gene Ontology [14]. Kyoto Encyclopedia of Genes and Genomes (KEGG http://www.genome.jp/kegg/pathway.html) pathway enrichment analysis was used to identify main functional and metabolic pathways involving DEGs. We used *P* value <0.01 as the cut-off criterion for the enrichment analysis which was conducted by the Database for Annotation, Visualization and Integrated Discovery (DAVID; version 2.1b, http://david.abcc.ncifcrf.gov/) online software [15].

### Transcription factors (TFs) and tumor-associated genes (TAGs) in DEGs

DEGs with the function of transcriptional regulation, namely, differentially expressed transcription factors were selected based on the TRANSFAC database (http://www.gene-regulation.com/pub/databases.html) [16]. According to the TSGene database (http://bioinfo.mc.vanderbilt.edu/TSGene/search.cgi) [17] and TAG database (http://www.binfo.ncku.edu.tw/TAG/) [18], we extracted the TAGs from DEGs, including tumor suppressor genes and oncogenes.

### Construction of PPI network and sub-network

The interaction pairs of DEGs were analyzed via online tool Search Tool for the Retrieval of Interacting Genes (STRING; version 9.0, http://string-db.org) [19], and interaction data were downloaded on June 27, 2014. Only the gene pairs which were recorded in database, and experimental validated, text-mined, or co-expressed were used and combined score >0.4 was set as the criterion of PPI. Then, the PPI network was constructed using Cytoscape (version 2.8, http://cytoscape.org) [20].

The sub-network analysis of PPI network was performed using the ClusterONE plug of Cytoscape [21]. The pathway enrichment analysis of the genes in the most significant sub-network was performed by using the DAVID online software [15].

## Results

### MM-induced DEGs in osteocytes

After analyzing the microarray data of control and co-culture groups, a total of 393 DEGs were screened out, including 167 down-regulated genes and 226 up-regulated genes in co-culture group.

### Functions and pathways enriched by DEGs

To explore the specific functions and pathways of DEGs, functional and pathway enrichment analyses were performed. The down-regulated genes were most significantly enriched in the functions of cardiovascular system development, circulatory system development (Table 1), and the pathways of protein digestion, absorption, and extracellular matrix (ECM)-receptor interaction pathway (Table 2). Especially, epidermal growth factor (*EGF*), sphingosine-1-phosphate receptor 1 (*S1PR1*), and Neuropeptide Y1 receptor (*NPY1R*) were involved in circulatory system development. Moreover, the up-regulated genes were most significantly enriched in the functions of regulation of response to stimulus and regulation of signaling function (Table 1), as well as the pathways of JAK-STAT signaling, nicotinate, and nicotinamide metabolism (Table 2).

**Table 1:** Function enrichment analysis of DEGs

Category	GO ID	GO term	Gene count	*P* value
Down	GO:0072358	Cardiovascular system development	35	2.22E-15
Down	GO:0072359	Circulatory system development	35	2.22E-15
Down	GO:0048731	System development	73	1.24E-14
Down	GO:0009653	Anatomical structure morphogenesis	55	1.44E-14
Down	GO:0031012	Extracellular matrix	19	5.70E-09
Down	GO:0005583	Fibrillar collagen	5	3.72E-08
Down	GO:0044420	Extracellular matrix part	12	5.72E-08
Down	GO:0005201	Extracellular matrix structural constituent	9	2.38E-08
Down	GO:0005178	Integrin binding	8	5.43E-07
Down	GO:0005198	Structural molecule activity	18	5.64E-06
UP	GO:0048583	Regulation of response to stimulus	67	5.57E-11
UP	GO:0023051	Regulation of signaling	59	2.48E-10
UP	GO:0010646	Regulation of cell communication	59	2.71E-10
UP	GO:0023052	Signaling	97	2.82E-10
UP	GO:0005615	Extracellular space	20	1.72E-03
UP	GO:0044421	Extracellular region part	24	2.83E-03
UP	GO:0031095	Platelet dense tubular network membrane	2	4.40E-03
UP	GO:0043565	Sequence-specific DNA binding	21	5.35E-05
UP	GO:0000982	RNA polymerase II core promoter proximal region sequence-specific DNA binding transcription factor activity	7	1.95E-04
UP	GO:0003700	Sequence-specific DNA binding transcription factor activity	25	3.44E-04

*GO* gene ontology; *ID* identifier; *DEGs* differentially expressed genes

**Table 2:** Pathway enrichment analysis of DEGs

Category	KEGG pathway	Gene count	*P* value
Down	Protein digestion and absorption	6	1.44E-04
Down	ECM-receptor interaction	6	1.88E-04
Down	Amoebiasis	6	6.24E-04
Down	Focal adhesion	8	7.86E-04
Down	Regulation of actin cytoskeleton	8	1.19E-03
Down	Axon guidance	6	1.74E-03
Down	Melanoma	4	5.42E-03
Down	Hypertrophic cardiomyopathy (HCM)	4	9.38E-03
Up	JAK-STAT signaling pathway	12	3.08E-06
Up	Nicotinate and nicotinamide metabolism	4	4.16E-04
Up	Calcium signaling pathway	8	5.14E-03
Up	VEGF signaling pathway	5	5.68E-03
Up	Phosphatidylinositol signaling system	5	6.34E-03
Up	Cytokine-cytokine receptor interaction	10	6.46E-03

*KEGG* Kyoto Encyclopedia of Genes and Genomes; *DEGs* differentially expressed genes

### Differentially expressed TFs and TAGs

The TFs and TAGs differentially expressed between control and co-culture groups were further extracted from DEGs. As shown in Table 3, four down-regulated TFs and 18 up-regulated TFs (e.g., Krüppel-like factors 4, *KLF4*; and IFN regulatory factor-8, *IRF8*) were discovered.

**Table 3:** Differentially expressed TFs and TAGs

Regulation	TF counts	TFs	TAG counts	Oncogenes	Tumor suppressor genes	Other TAGs
Down	4	TGFB1I1, NKX2-2, KLF5, ID2	16	RUNX1T1, PDGFRB, CD24	TPM1, SPTBN1,PRICKLE1, KLF5, IGFBP3, FAT4,EFNA1, DNAJB4, DAB2, CADM1	TGFB2, LRRC17, BAMBI
Up	18	SOX9, SOX7, NR3C2, MSX1, MAFF, KLF4, JUNB, IRF8, HOXB6, HMGA2, HES1, OXF1, FOXA2, ETV5, ETV4, ELK3, EGR3, EGR1	21	VEGFA, SPHK1, PDGFRA, JUNB, HMGA2, FOSL1, FGF5, ETV1, CCND1	SPRY2, SOX7, IRF8, IL24, HOPX, FOXA2, EPHB2, ENC1, EGR1, DUSP6	KLF4, EMP1

*TF(s)* transcription factor(s); *TAG(s)* tumor-associated gene(s)

Furthermore, 16 down-regulated TAGs and 21 up-regulated TAGs were screened out (Table 3). Among the down-regulated TAGs, there were 10 tumor suppressor genes and 3 oncogenes. Meanwhile, among the up-regulated TAGs, there were 10 tumor suppressor genes (e.g. *IRF8*) and 9 oncogenes.

### Construction of PPI network and sub-network

**Fig. 1:**
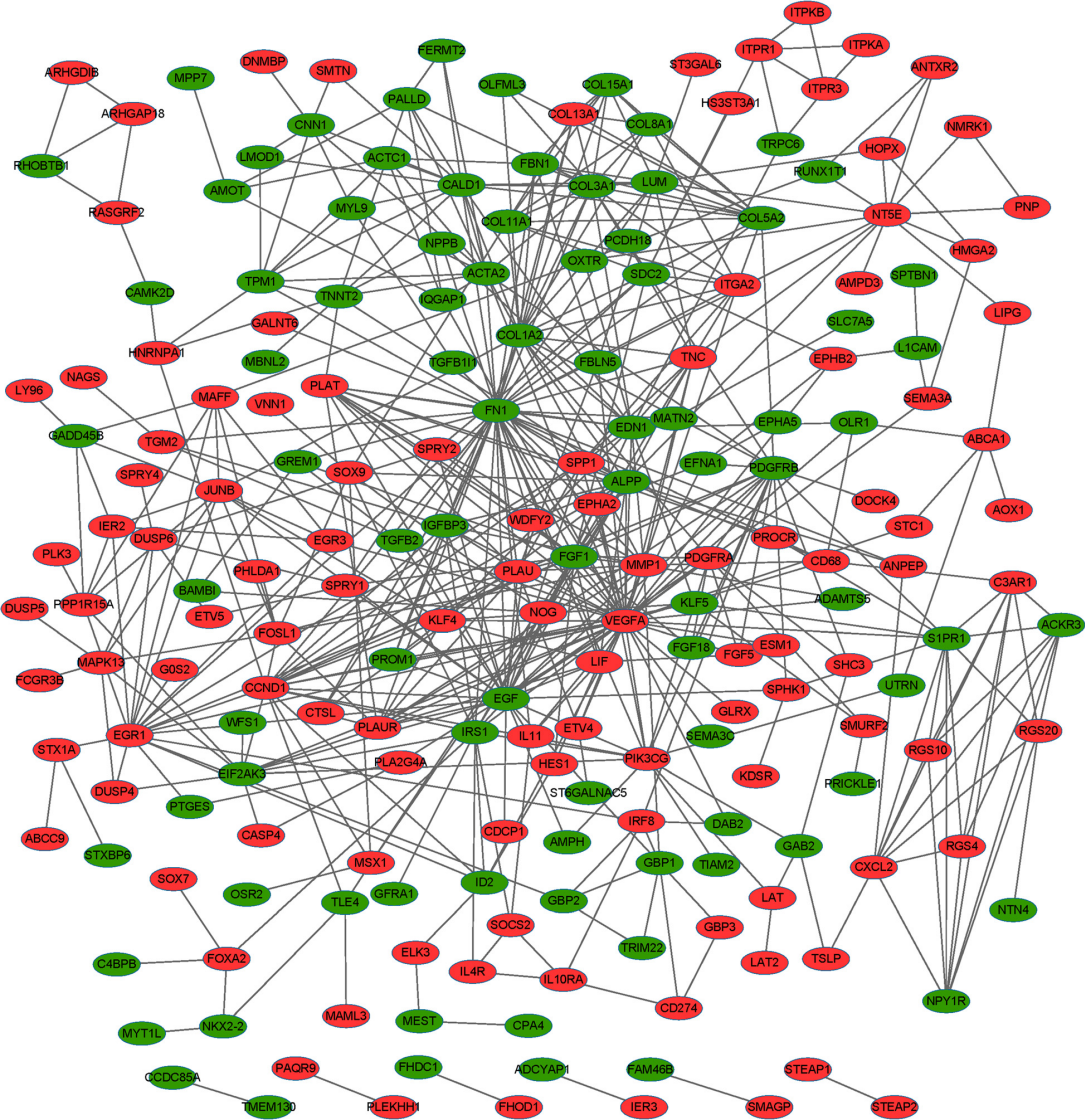
Protein-protein interaction (PPI) network of differentially expressed genes (DEGs). *Red* and *green nodes* represent up-regulated genes and down-regulated genes, respectively

Interactions between DEGs were identified using STRING software. By integrating DEG pairs with combined score >0.4, a PPI network was built (Fig. 1), consisting of 89 down-regulated genes and 113 up-regulated genes. A total of 10 DEGs had connectivity degree larger than 15, e.g., EGF (degree = 31) and early growth response 1 (EGR1, degree = 19). Besides, EGF could interact with EGR1 in the PPI network.

**Fig. 2:**
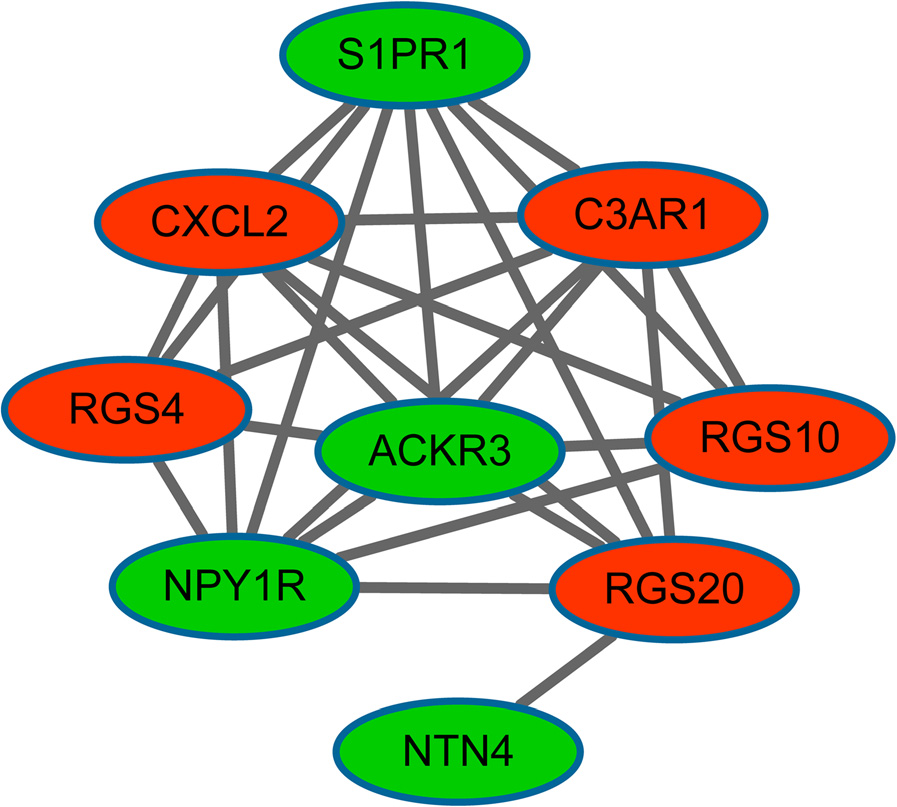
Sub-network in the protein-protein interaction (PPI) network of differentially expressed genes (DEGs). *Red* and *green nodes* stand for up-regulated genes and down-regulated genes, respectively

Moreover, a sub-network was obtained from PPI network (*P* value = 0.0004.3) (Fig. 2), consisting of nine significant DEGs like S1PR1, complement-3a receptor1 (C3AR1), and NPY1R. In the sub-network, S1PR1, C3AR1, and NPY1R had interactions with each other. Furthermore, the up-regulated *C3AR1*, as well as the down-regulated *S1PR1* and *NPY1R* in the sub-network were enriched in the neuroactive ligand-receptor interaction pathway (Table 4).

**Table 4:** Pathways enriched by the genes in sub-network

KEGG pathway	Gene count	*P* value	Genes
Neuroactive ligand-receptor interaction	3	1.42E-02	C3AR1, S1PR1, NPY1R

*KEGG* Kyoto Encyclopedia of Genes and Genomes

## Discussion

To gain insight into the molecular mechanisms of the myeloma-induced osteocyte death, gene expression profiles in osteocytes co-cultured with or without myeloma cells were systematically analyzed here. A total of 226 up-regulated genes and 167 down-regulated genes were identified. Then, PPI network was constructed, and sub-network was identified.

TFs like *KLF4* and *IRF8* were up-regulated in osteocytes co-cultured with myeloma cells in comparison with osteocytes cultured alone. Previous study reported that *KLF4* expression is essential for blocking cell cycle and increasing the resistance of MM cells to alkylating agents [22]. As one of the Yamanaka reprogramming factors and TFs, *KLF4* can promote the expression of autophagy-related genes [23]. In addition, silencing of the interferon consensus sequence-binding protein (*ICSBP/IRF8*) gene may be induced by DNA methylation or other mechanisms and correlates with the malignant phenotype of MM [24]. In our study, *KLF4* and *IRF8* were up-regulated TFs, and *IRF8* was a tumor suppressor gene. These suggested that *KLF4* and *IRF8* might play a role in osteolysis in MM patients.

In this study, EGF and EGR1 had high connectivity degrees in PPI network. A heparin-binding factor in EGF family, amphiregulin (*AREG*) is overexpressed in primary myeloma cells and can promote growth of bone marrow stromal cell [25]. *EGR-1* induces apoptosis in MM via interacting with *JUN*, and decreased *JUN/EGR-1* can enhance resistance of MM cells to bortezomib [26]. Functional enrichment indicated that *EGF* was involved in circulatory system development. It has been reported that the substances of the circulatory system can induce the apoptosis of tumor cells [27]. EGF could interact with EGR1 in the PPI network, indicating that *EGF* and *EGR1* might be involved in osteocytes apoptosis induced by MM cells through interacting with each other.

Enrichment analysis suggested that *S1PR1*, *C3AR1*, and *NPY1R* in sub-network were enriched in the pathway of neuroactive ligand-receptor interaction, meanwhile *S1PR1* and *NPY1R* were involved in the function of circulatory system development. S1PR1 is a G-protein-coupled receptor which binds to the bioactive signaling molecule sphingosine 1-phosphate (S1P) [28]. It is reported that *S1PR1* and *S1PR2* regulate osteoclast precursor migration between the bone marrow cavities and the circulation [29]. It is shown that component 3 (C3A) and its receptor C3AR play a role in osteoclast formation [30, 31], implying a potential role of *C3AR* in osteocyte death. Thus, *S1PR1* and *C3AR1* may promote the death of osteocytes, and this is consistent with the finding of Giuliani et al. [10]. *NPY1R* is a receptor of neuropeptide Y (*NPY*), which is a neurotransmitter. There is evidence that *NPY* regulates bone homeostasis via actions in peripheral tissues [32]. *NPY1R* is the only Y receptor which is robustly expressed in bone marrow stromal cells [33] and osteoblasts [34], implying a direct role of *NPY1R* in bone remodeling. In addition, the absence of peripheral Y1 receptor will lead to pronounced anabolic effects on the bone [35]. Here, *NPY1R* was significantly down-regulated in osteocytes co-cultured with myeloma cells, inhibiting bone remodeling. In the sub-network, S1PR1, C3AR1, and NPY1R had interactions with each other, suggesting that *S1PR1*, *C3AR1*, and *NPY1R* might function in osteocytes apoptosis induced by MM cells via interactions.

## Conclusions

In conclusion, a total of 393 DEGs were identified between osteocytes co-cultured with and without myeloma cells, and *KLF4*, *IRF8*, *EGF*, *EGR1*, *S1PR1*, *C3AR1*, and *NPY1R* might be involved in osteocyte cell apoptosis induced by MM cells. Here, we studied the potential molecular mechanism of the increased osteocyte death, which may provide a theoretical basis for understanding and treatment of osteolysis in MM patients. However, the present study analyzed gene expression data generated from not primary cells isolated from myeloma patients and healthy controls but cell lines, and the sample size was small. Thus, confirmation of results by quantitative real-time polymerase chain reaction (qRT-PCR) in cell lines followed by the investigation of the roles and functions of candidate genes in cells isolated from MM patients and healthy controls is still needed.

## Abbreviations

DEGs, differentially expressed genes; DKK1, Dickkopf 1; ECM, extracellular matrix; EGF, epidermal growth factor; KEGG, Kyoto Encyclopedia of Genes and Genomes; MM, multiple myeloma; NPY1R, neuropeptide Y1 receptor; PPI, protein-protein interaction; RMA, robust multi-array average; S1P, sphingosine 1-phosphate; S1PR1, sphingosine-1-phosphate receptor 1; STRING, Search Tool for the Retrieval of Interacting Genes; TAG, tumor-associated gene; TAGs, tumor-associated genes; TFs, transcription factors; TRAIL, tumor necrosis factor-related apoptosis-inducing ligand

## References

[1] Campa D, Martino A, Varkonyi J, Lesueur F, Jamroziak K, Landi S, Jurczyszyn A, Marques H, Andersen V, Jurado M (2015). Risk of multiple myeloma is associated with polymorphisms within telomerase genes and telomere length. Int J Cancer.

[2] Howlader N, Noone A, Krapcho M, Neyman N, Aminou R, Altekruse S, Kosary C, Ruhl J, Tatalovich Z, Cho H (2012). SEER cancer statistics review, 1975–2009 (vintage 2009 populations).

[3] Rajkumar SV (2011). Multiple myeloma: 2011 update on diagnosis, risk‐stratification, and management. Am J Hematol.

[4] Bergsagel PL, Mateos M-V, Gutierrez NC, Rajkumar SV, San Miguel JF (2013). Improving overall survival and overcoming adverse prognosis in the treatment of cytogenetically high-risk multiple myeloma. Blood.

[5] Crockett JC, Rogers MJ, Coxon FP, Hocking LJ, Helfrich MH (2011). Bone remodelling at a glance. J Cell Sci.

[6] Tian E, Zhan F, Walker R, Rasmussen E, Ma Y, Barlogie B, Shaughnessy JD (2003). The role of the Wnt-signaling antagonist DKK1 in the development of osteolytic lesions in multiple myeloma. N England J Med.

[7] Kocemba KA, Groen R, Van Andel H, Kersten MJ, Mahtouk K, Spaargaren M, Pals ST (2012). Transcriptional silencing of the Wnt-antagonist DKK1 by promoter methylation is associated with enhanced Wnt signaling in advanced multiple myeloma. PLoS One..

[8] Kawano Y, Ueno S, Abe M, Kikukawa Y, Yuki H, Iyama K, Okuno Y, Mitsuya H, Hata H (2012). TRAIL produced from multiple myeloma cells is associated with osteolytic markers. Oncol Rep.

[9] Cao H, Zhu K, Qiu L, Li S, Niu H, Hao M, Yang S, Zhao Z, Lai Y, Anderson JL (2013). Critical role of AKT protein in myeloma-induced osteoclast formation and osteolysis. J Biol Chem.

[10] Giuliani N, Ferretti M, Bolzoni M, Storti P, Lazzaretti M, Dalla Palma B, Bonomini S, Martella E, Agnelli L, Neri A (2012). Increased osteocyte death in multiple myeloma patients: role in myeloma-induced osteoclast formation. Leukemia..

[11] Gautier L, Cope L, Bolstad BM, Irizarry RA (2004). affy—analysis of Affymetrix GeneChip data at the probe level. Bioinformatics.

[12] Irizarry RA, Hobbs B, Collin F, Beazer‐Barclay YD, Antonellis KJ, Scherf U, Speed TP (2003). Exploration, normalization, and summaries of high density oligonucleotide array probe level data. Biostatistics..

[13] Haynes W. Student’s t-test. Encyclopedia of systems biology. New York; Springer; 2013; p. 2023–5.

[14] Ashburner M, Ball CA, Blake JA, Botstein D, Butler H, Cherry JM, Davis AP, Dolinski K, Dwight SS, Eppig JT (2000). Gene Ontology: tool for the unification of biology. Nat Genet.

[15] Huang DW, Sherman BT, Lempicki RA (2008). Systematic and integrative analysis of large gene lists using DAVID bioinformatics resources. Nat Protocols.

[16] Matys V, Fricke E, Geffers R, Gößling E, Haubrock M, Hehl R, Hornischer K, Karas D, Kel AE, Kel-Margoulis OV (2003). TRANSFAC®: transcriptional regulation, from patterns to profiles. Nucleic Acids Res.

[17] Zhao M, Sun J, Zhao Z (2013). TSGene: a web resource for tumor suppressor genes. Nucleic Acids Res.

[18] Chen J-S, Hung W-S, Chan H-H, Tsai S-J, Sun HS (2013). In silico identification of oncogenic potential of fyn-related kinase in hepatocellular carcinoma. Bioinformatics..

[19] Franceschini A, Szklarczyk D, Frankild S, Kuhn M, Simonovic M, Roth A, Lin J, Minguez P, Bork P, von Mering C (2013). STRING v9. 1: protein-protein interaction networks, with increased coverage and integration. Nucleic Acids Res.

[20] Smoot ME, Ono K, Ruscheinski J, Wang P-L, Ideker T (2011). Cytoscape 2.8: new features for data integration and network visualization. Bioinformatics.

[21] Demchak B, Hull T, Reich M, Liefeld T, Smoot M, Ideker T, Mesirov JP (2014). Cytoscape: the network visualization tool for GenomeSpace workflows. F1000Research.

[22] Schoenhals M, Kassambara A, Veyrune J-L, Moreaux J, Goldschmidt H, Hose D, Klein B (2013). Krüppel-like factor 4 blocks tumor cell proliferation and promotes drug resistance in multiple myeloma. Haematologica.

[23] Riz I, Hawley TS, Hawley RG (2015). KLF4-SQSTM1/p62-associated prosurvival autophagy contributes to carfilzomib resistance in multiple myeloma models. Oncotarget..

[24] Tshuikina M, Jernberg-Wiklund H, Nilsson K, Oberg F (2008). Epigenetic silencing of the interferon regulatory factor ICSBP/IRF8 in human multiple myeloma. Exp Hematol.

[25] Mahtouk K, Hose D, Rème T, De Vos J, Jourdan M, Moreaux J, Fiol G, Raab M, Jourdan E, Grau V (2005). Expression of EGF-family receptors and amphiregulin in multiple myeloma. Amphiregulin is a growth factor for myeloma cells. Oncogene.

[26] Chen L, Wang S, Zhou Y, Wu X, Entin I, Epstein J, Yaccoby S, Xiong W, Barlogie B, Shaughnessy JD (2010). Identification of early growth response protein 1 (EGR-1) as a novel target for JUN-induced apoptosis in multiple myeloma. Blood..

[27] Kulcsár G (1997). Apoptosis of tumor cells induced by substances of the circulatory system. Cancer Biother Radiopharm.

[28] Maceyka M, Harikumar KB, Milstien S, Spiegel S (2012). Sphingosine-1-phosphate signaling and its role in disease. Trends Cell Biol.

[29] Ishii M, Kikuta J (2013). Sphingosine-1-phosphate signaling controlling osteoclasts and bone homeostasis. Biochimica et Biophysica Acta (BBA)-Molecular and Cell Biology of Lipids.

[30] Abe M, Hiura K, Wilde J, Shioyasono A, Moriyama K, Hashimoto T, Kido S, Oshima T, Shibata H, Ozaki S (2004). Osteoclasts enhance myeloma cell growth and survival via cell-cell contact: a vicious cycle between bone destruction and myeloma expansion. Blood..

[31] Ignatius A, Schoengraf P, Kreja L, Liedert A, Recknagel S, Kandert S, Brenner RE, Schneider M, Lambris JD, Huber‐Lang M (2011). Complement C3a and C5a modulate osteoclast formation and inflammatory response of osteoblasts in synergism with IL‐1β. J Cell Biochemistry.

[32] Sousa D, Herzog H, Lamghari M (2009). NPY signalling pathway in bone homeostasis: Y1 receptor as a potential drug target. Current Drug Targets.

[33] Lundberg P, Allison SJ, Lee NJ, Baldock PA, Brouard N, Rost S, Enriquez RF, Sainsbury A, Lamghari M, Simmons P (2007). Greater bone formation of Y2 knockout mice is associated with increased osteoprogenitor numbers and altered Y1 receptor expression. Journal of Biological Chemistry..

[34] Teixeira L, Sousa DM, Nunes AF, Sousa MM, Herzog H, Lamghari M (2009). NPY revealed as a critical modulator of osteoblast function in vitro: new insights into the role of Y1 and Y2 receptors. Journal of cellular biochemistry..

[35] Lee NJ, Nguyen AD, Enriquez RF, Doyle KL, Sainsbury A, Baldock PA, Herzog H (2011). Osteoblast specific Y1 receptor deletion enhances bone mass. Bone..

